# Tens-of-Grams Synthesis of β-NaLnF_4_ Upconversion Particles Using Fluorine Excess and Inverted Crucibles as the Sintering Device

**DOI:** 10.1021/acsomega.4c08889

**Published:** 2025-02-04

**Authors:** Haggeo Desirena, Jorge A. Molina-González, Mario Alan Quiroz-Juárez, Gonzalo Ramírez-García

**Affiliations:** † Centro de Investigaciones en Óptica A.C., A.P. 1-948, León, Guanajuato 37150 México; ‡ Biofunctional Nanomaterials Laboratory, Universidad Nacional Autónoma de México, Centro de Física Aplicada y Tecnología Avanzada, 3001, Boulevard Juriquilla, Querétaro 76000, México; § Universidad Nacional Autónoma de México, Centro de Física Aplicada y Tecnología Avanzada, 3001, Boulevard Juriquilla, Querétaro 76000, México

## Abstract

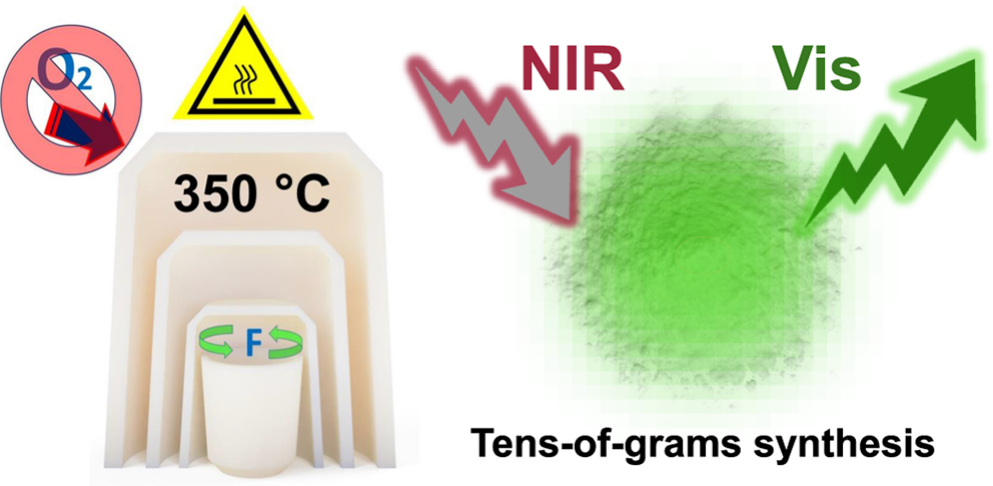

This work presents a robust method for the synthesis of pure β-NaLnF_4_ upconversion particles (Ln = rare-earths) on a tens-of-grams scale. The sintering process was improved by incorporating NH_4_HF_2_ as a fluorinating agent and by arranging three inverted crucibles in increasing sizes to mitigate heat dissipation. This synergistically reduced the sintering temperature from over 550 to 350 °C and decreased the heating time from several hours to just 30 min. This method offers several advantages: (i) prevents impurities and surface oxygen defects that disrupt upconversion frequency due to multiphoton relaxation; (ii) requires a simple experimental setup, eliminating the need for inert atmospheres, furnaces, or special reactors; (iii) avoids the use of organic solvents for separation and washing; (iv) allows modulation of particle size at the submicrometric scale based on sintering temperature and heating time; and (v) provides quantitative yields in the tens-of-grams scale. This strategy could enable the mass production and broad distribution of upconverting materials, which are crucial for developing a wide range of advanced products with enhanced performance, including sensors, contrast agents, solar cells, security printing, and light-emitting devices.

## 1 Introduction

Upconverting materials have garnered significant interest in various research fields due to their unique optical properties, including their ability to convert low-energy photons to high-energy ones. Some applications of upconverting materials include optical imaging, sensing, photoactivated therapies, drug delivery, energy conversion, catalysis, document authentication, and anticounterfeiting.^1−6^ β-NaYF_4_ (hexagonal crystalline phase) is one of the most efficient host lattices for upconverting materials (UC) phosphors activated under infrared (IR) excitation.^7,8^ The conventional methods for the preparation of this material include solid-state reaction, thermal decomposition, coprecipitation, microwave-assisted reactions, and solvothermal approaches.^9−13^ However, their broad applications are usually limited by the lack of protocols that yield large quantities of materials with homogeneous optical and structural properties. To date, the dominant method for producing UC nanoparticles primarily relies on wet chemical processes. These approaches involve several sequential steps and often require preparation exceeding 12 h. Despite their effectiveness, one notable drawback is the limited quantity of material produced per reaction batch. Solid-state reaction is the most common method used for the large-scale synthesis of β-NaLnF_4_. This reaction proceeds through a solid-state diffusion mechanism that typically requires temperatures in the range of 550–1000 °C. The described protocols need hours of thermal treatment, are carried out under a controlled atmosphere, and indiscriminately require a furnace to reach those temperatures.^14^ Therefore, it is necessary to develop novel strategies that reduce the complexity and energetic requirements of the current methods without compromising the optical properties, reaction yield, purity, and overall quality of the upconverting materials.

In practical terms, the efficiency of upconverting materials prepared through the solid-state reaction depends primarily on the temperature and duration of the sintering treatment.^15^ For instance, solid-state methods that use mixtures of lanthanide fluorides, carbonates, or nitrates as precursors typically require an annealing step at temperatures exceeding 600 °C for several hours to render pure β-NaLnF_4_ materials.^16,17^ In other works, successive heating steps (450 °C/6h and then 550 °C/6h) are further necessary to obtain materials with the desired crystalline phase.^18^ The reduction of the sintering temperature for the formation of β-NaLnF_4_ is a critical parameter, as it compromises the purity of the crystalline phase. Therefore, the use of elevated temperatures remains a technical limitation that must be overcome to develop methods that enable large-scale production of upconverting particles. Moreover, most of the reported methods also require an excess of fluorine precursors. Fluorination is a strategy to improve the upconversion efficiency of lanthanide-doped NaYF_4_ materials because this process removes oxygen or other impurities associated with nonradiative transitions.^19^ For example, a synthesis under an HF/Ar gas stream has been performed for 20 h during repeated heating steps at 550 °C.^15^ Another method explored the addition of NH_4_HF_2_ under an HF gas atmosphere for the solid-state synthesis of β-Na(Y_1.5_Na_0.5_)F_6_:Tm^3+^ at 700 °C for 6 h.^20^ Elsewhere, cubic α-NaYF_4_ nanoparticles were mixed with ZnF_2_ or SnF_2_ and sintered at 590 °C, resulting in the phase transition to submicrometric β-NaYF_4_ particles with improved luminescence.^21^ Given the complexity of these procedures, simpler reaction conditions and lower preparation temperatures that reduce energy consumption and simplify the equipment for synthesis are highly demanded for spreading the production of upconverting materials.

This work reports a simple, robust, and easily replicable methodology for the time-saving synthesis of large quantities (more than 20 g per batch) of pure β-NaLnF_4_ (Ln= lanthanides) submicrometric particles. The addition of NH_4_HF_2_ as a fluorinating agent reduces the annealing temperature to levels that are easily reached on a hot plate. This agent promotes the integration of the reagents in the molten phase, thus enhancing the homogeneity of the products as in wet chemistry but with the advantages of a solid-state reaction. Moreover, the incorporation of an inverted-crucible system decreased the heat dissipation, thus shortening the heating step duration. Therefore, the suggested approach involves a straightforward solid-state reaction that avoids the use of a furnace and inert atmosphere generation system, as in conventional methods. This system also minimizes the free exchange of gases between the sample and the atmosphere, improving the upconverting emissions due to the prevention of impurities and surface oxygen defects that can affect the luminescence.

## 2 Experimental Section

### 2.1 Materials

*Reagents*: sodium fluoride (NaF) 99.99%, yttrium fluoride (YF_3_) 99.99%, ytterbium fluoride (YbF_3_) 99.99%, erbium fluoride (ErF_3_) 99.99%, thulium fluoride (TmF_3_) 99.99%, and holmium fluoride (YF_3_) 99.99% were purchased from Alfa Aesar. Ammonium bifluoride (NH_4_HF_2_) and hydrofluoric acid (HF) were provided by Sigma-Aldrich. Methanol was purchased from a local provider.

### 2.2 Synthesis of NaYF_4_:Yb^3+^,Er^3+^ Particles

**Figure 1:**
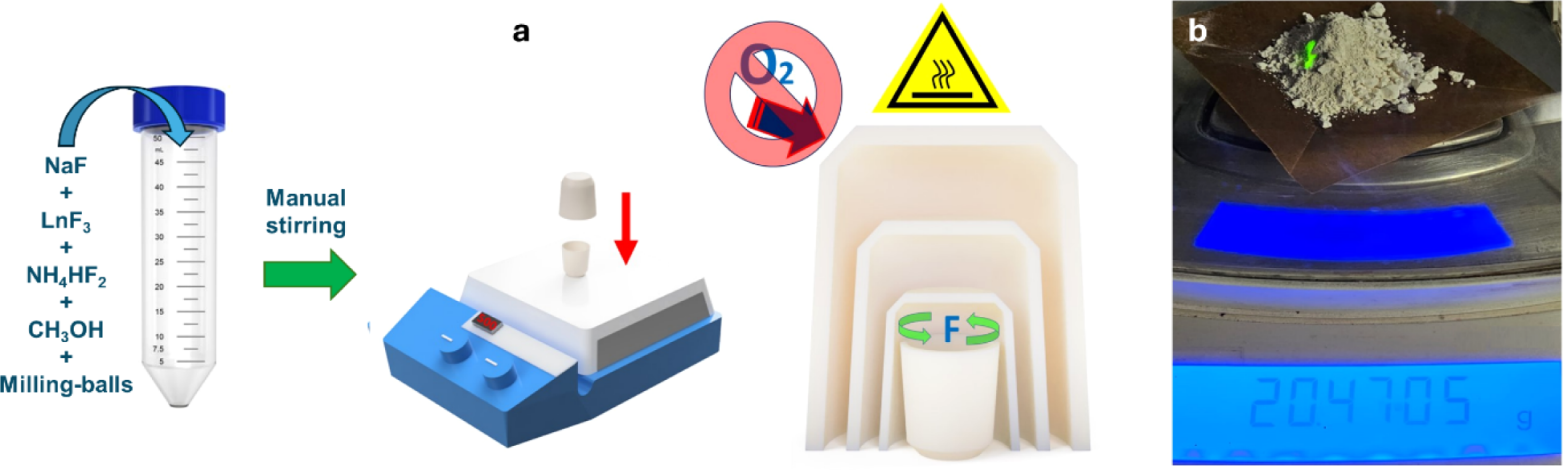
(a) Schematic illustration of the synthesis of NaLnF_4_ particles with the inverted-crucible sintering device and (b) Tens-of-grams scale production of UC particles on a hot plate. The arrow points to the emission from an area irradiated with a 975 nm portable laser.

β-NaYF_4_:Yb^3+^,Er^3+^ particles were synthesized as illustrated in Figure 1a. The standard method for the synthesis of 4 g of upconverting particles consists of the following general steps: 1) NaF and LnF_3_ (Ln = Y, Yb, Er, Tm, or Ho) precursors were weighed according to Table 1 and blended in a beaker. 2) The raw materials were mixed with 4 g of 3 mm alumina grinding balls and 3.5 mL of methanol in a centrifuge tube. The tube was vigorously shaken with hands for 5 min to promote the particle homogenization. 3) 1 mL of the fluorinating agent (NH_4_HF_2_ or HF) was added to the mixture and shaken again for 5 min. Control experiments were performed without the fluorinating reagent. 4) The mixture was transferred to a crucible and dried at 80 °C for 3 h. 5) Materials were sintered on a hot plate in an air atmosphere at temperatures in the 350–500 °C range for 15, 30, 60, 90, 120, or 180 min. Samples were directly heated in the uncovered crucible, or the crucible was covered with three inverted crucibles stacked in increasing size (20, 50, and 200 mL) as shown in Figure 1a.

**Table 1:** Weight Calculations for the Synthesis of Upconverting Materials

		Material weight (g)		
Precursor	Molecular weight (g/mol)	NaY_0.8_F_4_:Yb_0.18_, Er_0.02_	NaY_0.8_F_4_:Yb_0.195_, Tm_0.005_	NaY_0.8_F_4_:Yb_0.18_, Ho_0.02_
NaF	41.98	0.840	0.840	0.840
YF_3_	145.9	2.334	2.334	2.334
YbF_3_	230.04	0.828	0.897	0.828
ErF_3_	224.254	0.090	0	0
TmF_3_	225.93	0	0.023	0
HoF_3_	221.93	0	0	0.089

### 2.3 Instrumentation

The crystalline structure of the samples was characterized using an X-ray diffractometer (XRD) from Bruker Instruments (D2 Phaser) equipped with Cu Kα radiation at 1.54184 Å. The recorded XRD spectra were obtained from 15° to 80° 2θ range with increments of 0.02° and a swept time of 0.5 s. The morphology of the samples was analyzed by scanning electron microscopy (SEM) using a JEOL JSM 7800F microscope. Raman spectra were collected with a Bruker Raman Senterra Spectrometer, which features an integrated microscope and a 785 nm infrared laser. The emission spectra were recorded by exciting the upconverting samples using an RLTMDL-975–2W infrared laser diode (LD) centered at 975 nm from a Roithner Laser. The signal emitted was focused onto an SP-2357 spectrograph from Acton Research and detected by an R955 Hamamatsu photomultiplier tube (PMT). The decay profile (lifetime) corresponding to the emission centered at 545 nm was recorded by pulsing the laser with a frequency of 100 Hz with a TDS 3025B Teledyne oscilloscope. The decay curves were fitted to a single exponential of the form ***I*_*t*_**=***I*_0_*e***^(-*t/τ*)^, where ***I*_*t*_** and ***I*_*0*_** are the luminescence intensities at times t and 0, respectively, and τ is the luminescence lifetime.

## 3 Results and Discussion

Figure 1a illustrates the procedure for the synthesis of NaLnF_4_. First, proper quantities of NaF, LnF_3_ (Ln= Yb, Y, Er, Tm, Ho), methanol, and milling balls were vigorously stirred by hand in a plastic centrifuge tube. Then, three schemes of heating on a hot plate in an air atmosphere were evaluated: (i) samples were directly heated in a crucible, (ii) the crucible was covered with three inverted crucibles (20, 50, and 200 mL) as shown in Figure 1a, and (iii) NH_4_HF_2_ was added during the milling process, followed by sintering with the inverted-crucible scheme as in (ii). The inverted crucibles were stacked in increasing size mode with multiple purposes, including the prevention of heat dissipation, the promotion of a faster temperature increase due to the greenhouse effect, and a physical barrier that minimizes oxygen exchange between the sample and the surrounding atmosphere. The reported method demonstrates the potential for easy scalability by increasing the container’s capacity and extending reaction time. Figure 1b provides an overview of the results, showing a 20 g batch of UC particles prepared on a hot plate. Compared to earlier protocols for the mass production of NaYF_4_,^22−24^ the approach presented here reduces the waste of organic solvents and chemical compounds during synthesis, while also improving reaction yield through a faster and more streamlined preparation process. In all cases, the temperature of the hot plate was brought to a fixed temperature in the 350–500 °C range, and sintering periods between 15 and 120 min were evaluated. More specific details are described in the Section 2.

**Figure 2:**
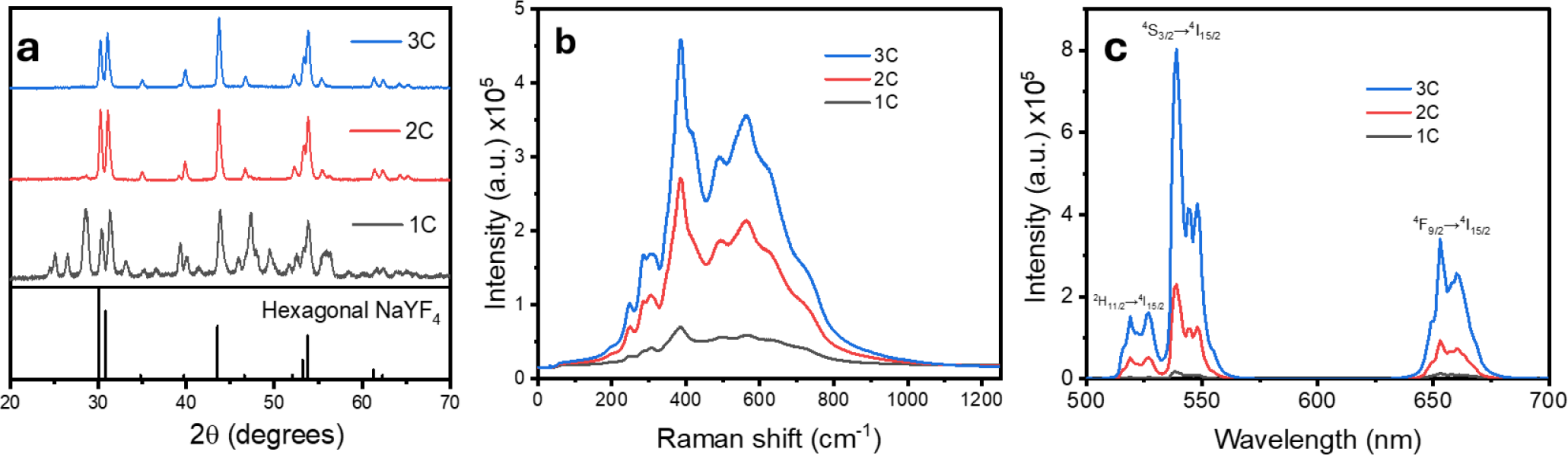
Effect of the number of crucibles on (a) the X-ray diffraction patterns, (b) the Raman spectra, and (c) the emission spectra of NaYF_4_:Yb,Er particles.

To study the efficiency of the synthesis device, the heating step was carried out using 1, 2, or 3 inverted crucibles arranged in increasing order of size. For this purpose, the reaction temperature was set at 500 °C, and the time at 60 min. The results are shown in Figure 2. In general, the use of three crucibles allowed for the synthesis of materials with a consolidated crystalline structure and higher emission intensity compared to the materials obtained with one or two crucibles. Initially, the XRD patterns revealed a pure hexagonal crystalline phase when using 3 crucibles, while precursor residues were observed with two crucibles, and an amorphous material when using one crucible. Similar results were obtained through Raman spectroscopy, where the sample synthesized with three crucibles exhibited a more intense signal, indicating a purer crystalline phase. This is consistent with the emission profiles, where the overall intensity follows the order 1C < 2C < 3C. Each of these parameters, as well as their implementation to optimize synthesis times and reaction temperature, is discussed in more detail in the following sections.

**Figure 3:**
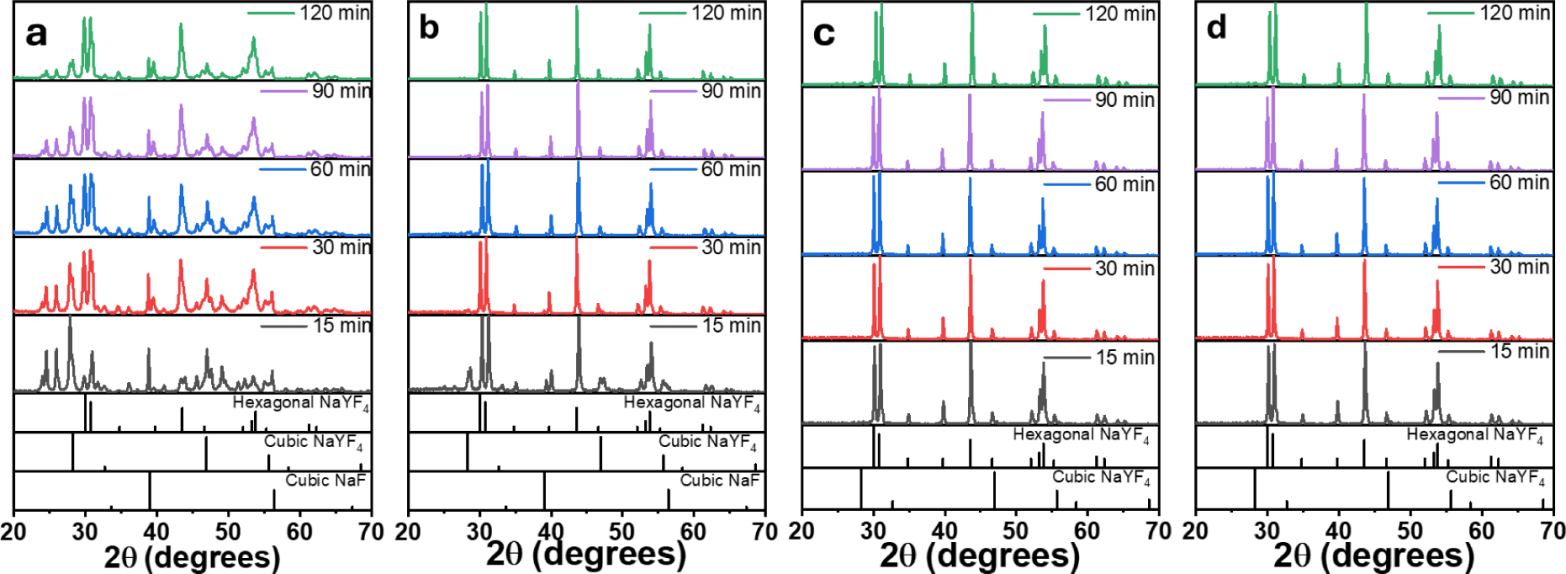
XRD patterns of particles sintered at 500 °C on a hot plate in (a) open crucible, (b) inverted-crucible device, inverted-crucible device upon addition of (c) NH_4_HF_2_ and (d) HF as fluorinating agents.

The influence of the three sintering schemes on the crystalline phase of the produced materials was evaluated through XRD. X-ray diffractograms presented in Figure 3a correspond to materials obtained in an open crucible system, where diffraction peaks are indexed to a mixture of cubic (α) and hexagonal (β) phases of NaYF_4_. The cubic phase shows a decrease in peak intensities, while the hexagonal phase becomes predominant with increasing reaction time. However, additional cubic NaF and other precursors such as LnF_3_ also remain evident at 24.81°, 26.21°, and 28.07°, which indicates that the temperature is still too low for all the precursors to fully react. Binary mixtures of the α-NaF and β-NaYF_4_ phases have also been reported elsewhere with methods in which the low temperature is responsible for unreacted precursors.^25−27^ To avoid heat dissipation, the crucible containing the precursors was encased within the inverted crucibles. As shown in Figure 3b, this method promotes the formation of the hexagonal crystalline phase upon annealing periods of >15 min, as evidenced by the heightened intensity of the corresponding peaks. In addition, peaks related to LnF_3_ were not observed. However, cubic phases corresponding to NaF and NaYF_4_ gradually vanish until reaching 120 min. This implies that longer reaction times are required to attain more favorable conditions for obtaining a material with a pure β-NaYF_4_ phase.

In the typical solid-state reaction methods, a fluorine precursor is often used in excess for the preparation of β-NaYF_4_.^14^ These conditions are employed to shift the reaction of the LnF_3_–NaF reagents toward the products with the desired crystalline phase. In the present work, NH_4_HF_2_ was used as a fluorinating agent in combination with the inverted-crucible scheme for synthesis. The XRD patterns of the resulting products are shown in Figure 3c, in which the hexagonal phase of NaYF_4_ becomes predominant at a shorter reaction time. After 15 min of reaction, the signal corresponding to cubic NaF and NaYF_4_ is scarcely observed and totally disappears above 30 min, indicating a full conversion to the desired hexagonal phase. Considering the melting point of NH_4_HF_2_ (126 °C), this precursor promotes the integration of the reagents in the molten phase, thus enhancing the homogeneity of the products as in wet chemistry but with the advantages of a solid-state reaction. Furthermore, the reaction of NaF, LnF_3_ and NH_4_HF_2_ is highly exothermic, which indicates that the formation of NaLnF_4_ could start during the mixing process and that the reaction can occur thoroughly in less time. The excess fluorinating agent is removed from the reaction mixture once the boiling point is reached (240 °C). This innovative strategy provides a fluorine-rich environment since it maintains F^–1^ ions circulating inside the inverted crucibles. Figure 3d provides valuable XRD data to understand the impact of another fluorinating agent (HF) on the crystalline phase of the reaction products. It can be easily observed that HF also works as an effective fluorinating agent, as well as NH_4_HF_2_. These results demonstrate the robustness of the method, and the systematic exploration of these modifications holds great promise for optimizing the sintering conditions.

**Figure 4:**
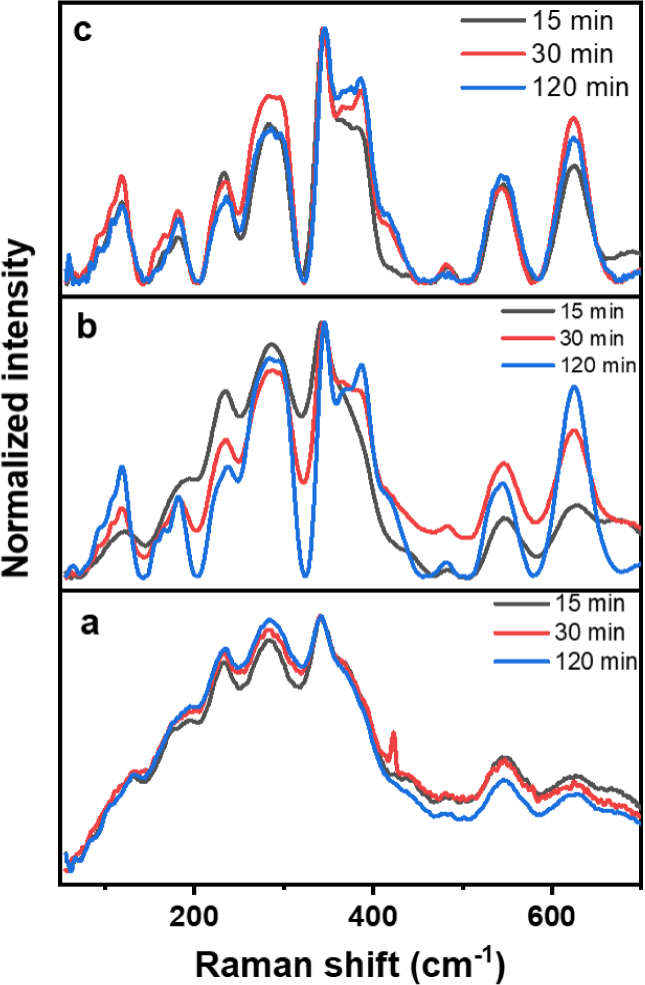
Raman spectra of representative samples synthesized at 500 °C with (a) open crucible, (b) inverted-crucible device, and (c) inverted-crucible device assisted by NH_4_HF_2_.

Raman spectroscopy demonstrated that the reported method not only allows favorable conditions for the development of the hexagonal NaYF_4_ phase but also fosters efficient crystallization. Raman spectra shown in Figure 4 are consistent with the presence of the hexagonal NaYF_4_ phase, as described in the literature.^28−32^ However, the Raman spectra of products obtained with an open crucible (Figure 4a) exhibit a broad band with overlapping peaks, which suggests a low degree of crystallinity. Meanwhile, analysis of products obtained through the inverted-crucible system (Figure 4b) resulted in narrower bands that indicate improved crystallinity compared to materials obtained with an open crucible. The bands in the Raman spectra of materials obtained through the inverted-crucible system upon addition of NH_4_HF_2_ as a fluorinating agent (Figure 4c) resulted even narrower and more intense, indicating a substantial enhancement in crystallinity in comparison to methods in which NH_4_HF_2_ was not added. In all three sample groups, the central position of each band remains constant. However, the decrease in bandwidth in Figure 4c directly correlates with the improved crystallization,^33,34^ which was enabled by the inverted-crucible system. The Raman spectra shown in Figure 4c reveal a series of peaks between 90 and 700 cm^–1^, corresponding to the signature of hexagonal β-NaYF_4_:Yb^3+^,Er^3+^ upconverting materials, as reported in other studies.^35,36^ In contrast, the spectra of samples obtained without inverted crucibles show only indistinct signals. The two broad peaks centered above 500 cm^–1^ for the materials synthesized using the inverted crucible system indicate high phonon energy, making these materials excellent hosts for upconversion emission mechanisms. This outcome underscores the significant advantages offered by the reported method.

**Figure 5:**
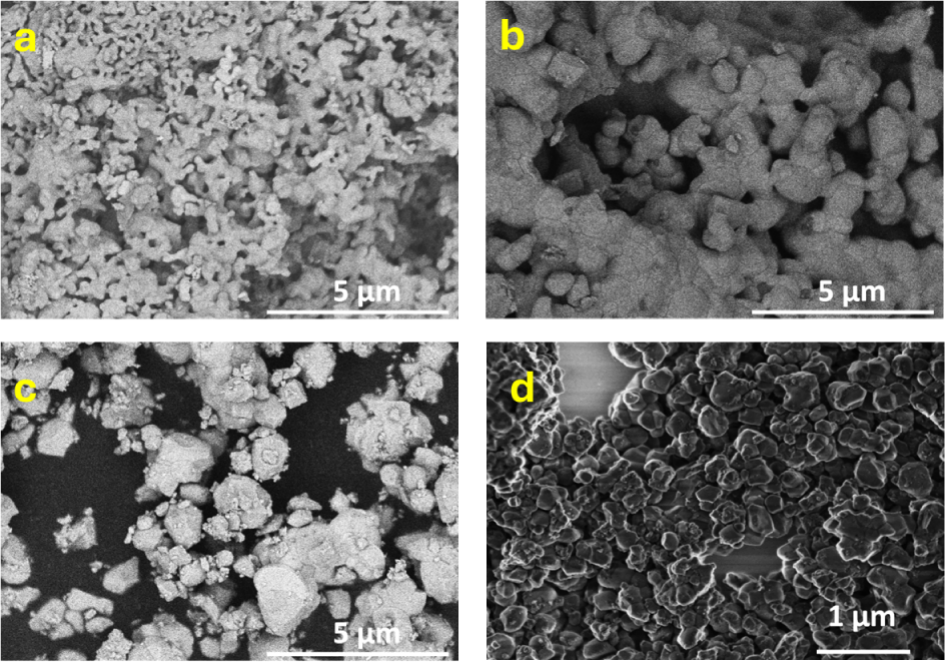
SEM images of the samples synthesized with a NH_4_HF_2_-assisted inverted-crucible device for (a) 15, (b) 60, (c) 120 min at 500 °C, and (d) 3h at 350 °C.

Figure 5a allows us to verify that larger synthesis periods with the inverted-crucible system assisted by NH_4_HF_2_ at **500 °C** induce a progressive coalescence of the resulting particles. After 15 min, submicrometric particles are formed, while after 120 min, these particles aggregate to reach dimensions over 2 μm. The coalescence process is accompanied by simultaneous crystallization and gradual unification of the hexagonal phase of NaYF_4_ as shown by XRD and Raman spectroscopy.

Using the inverted-crucible system strategy assisted by NH_4_HF_2_, the synthesis of particles was also evaluated at sintering temperatures as low as 350 °C to prevent coalescence. However, the reaction time had to be extended to 3 h to observe the proper crystalline structure of the materials, and thus, efficient photoluminescence processes. Figure 5d shows the scanning electron micrograph of the resulting material, demonstrating the formation of particles with less size dispersion, ranging from 50 to 230 nm. This observation also demonstrates the possibility of modulating the particle size by adjusting the temperature and duration of the sintering step.

**Figure 6:**
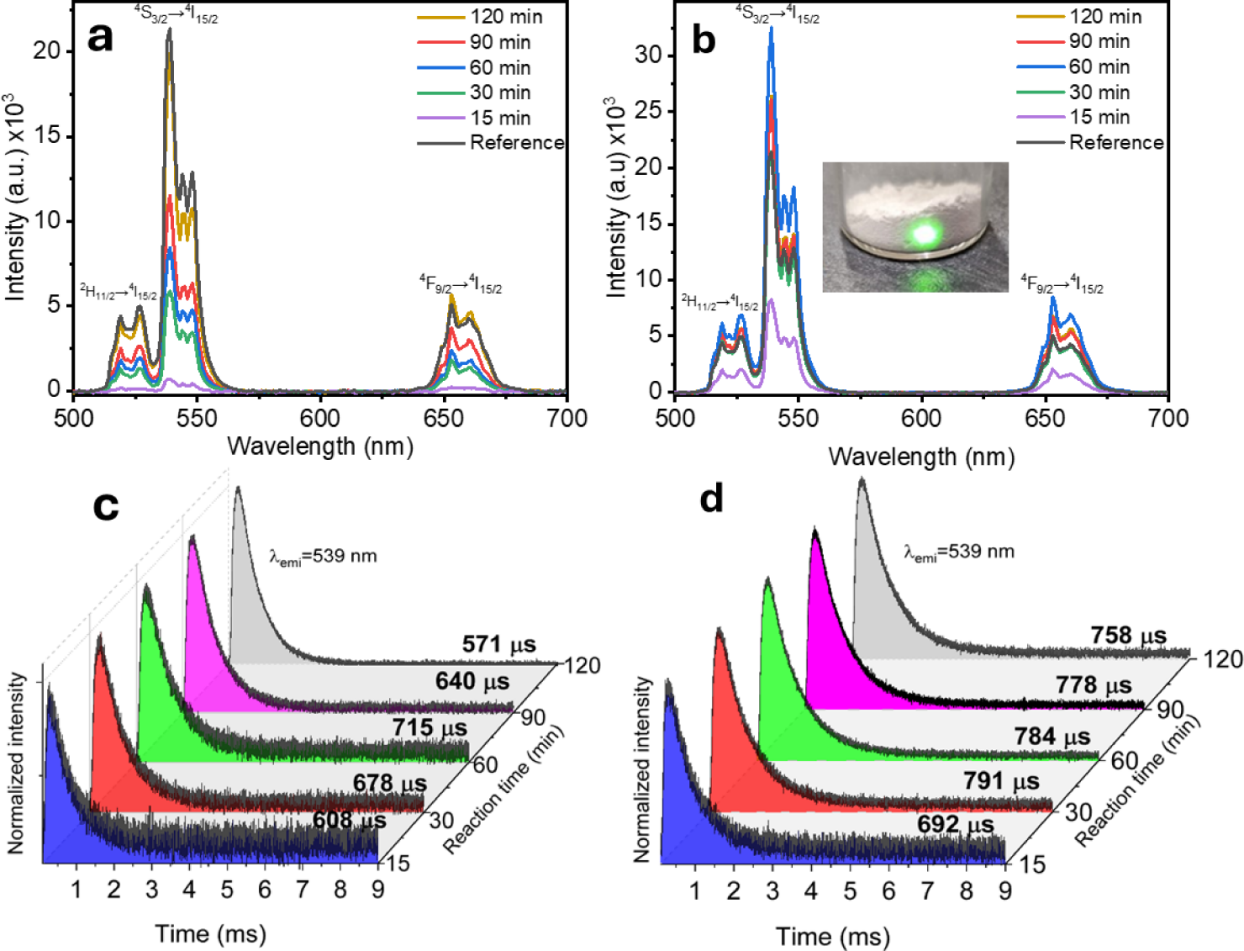
PL emissions and decay curves of NaYF_4_:Yb^3+^,Er^3+^ synthesized at 500 °C through (a,c) the inverted-crucible device and (b,d) the inverted-crucibles device assisted by NH_4_HF_2_. The inset in (b) show the photography of sample exhibiting high UC emission under 975 nm.

Photoluminescence (PL) properties of the resulting upconverting materials are shown in Figure 6. Upon excitation with a 975 nm/100 mW laser, the samples synthesized with the inverted-crucible system presented intense green and red upconversion emissions that reached levels similar to those of commercially available NaYF**_4_**:Yb^3+^,Er^3+^ microparticles (Sigma-Aldrich), even when a fluorinating agent was not added (Figure 6a). The three distinctive bands centered at 527, 539, and 653 nm are associated with ^2^H_11/2_ → ^4^I_15/2_, ^4^S_3/2_ → ^4^I_15/2_, ^4^F_9/2_ → ^4^I_15/2_ transitions of Er^3+^ respectively. Upconversion emission signals were not detected under similar conditions for materials obtained with the open crucible approach upon annealing for 15, 30, and 60 min, while weak emissions are hardly noticeable in samples annealed for 90 and 120 min (results not shown). This is well correlated with the diffraction peaks observed in Figure 3b, which are associated with the presence of unreacted LnF_3_ and NaF precursors, as well as the less efficient α-NaYF_4_:Yb^3+^,Er^3+^. Heat dissipation is significant when an open crucible is used, while the inverted-crucible system introduces a more efficient heat transfer mechanism via thermal convection. The incorporation of a fluorinating agent demonstrated a significant impact on enhancing the upconversion emissions, and the UC emission of materials obtained with the inverted-crucible system increased with longer reaction times. For instance, the overall intensity was enhanced up to 360% when the sintering time increased from 15 to 60 min, leading to a strong UC emission that is derived from the exclusive NaYF_4_:Yb^3+^,Er^3+^ hexagonal phase (Figure 6b). The UC emission of the optimized sample was enhanced by up to 100% compared to the reference consisting of commercially available NaYF**_4_**:Yb^3+^,Er^3+^ microparticles (Sigma-Aldrich). The combination of the inverted-crucible device and the addition of a fluorinating agent enables the sample to reach a higher temperature, reducing reaction time and thereby enhancing the photoluminescent signals. The inset in Figure 6b shows such a UC signal, which can be easily observed under ambient room light with a power excitation of 25 mW.

Figure 6c,d shows the photoluminescence decay time of samples obtained through the inverted-crucible system with or without NH_4_HF_2_, respectively. Figure 6c shows a progressive increase in decay time from 608 to 715 μs as the reaction time lengthens from 15 to 60 min. However, it decreases to 571 μs for 120 min. Since the concentration of Yb^3+^ and Er^3+^ was fixed in the studied samples, the increase in decay times could be derived from their interaction with the host material. In this regard, the initial increase in decay time is attributed to the enhanced crystallization, as evidenced in Raman analysis. However, Yb^3+^ ions can reduce the cluster formation and the consequent energy transfer between Er^3+^ par ions, leading to an increase in decay time.^37^ In the presence of the fluorinating agent, decay time increased from 692 to 791 μs for 15 and 30 min and subsequently decreased to 758 μs for 120 min, as shown in Figure 6d. Decay time in the presence of NH_4_HF_2_ is larger, indicating a lower effect of concentration quenching. It has been demonstrated that the hexagonal NaYF_4_ phase displays significant cationic disorder, where Na^+^ and Y^3+^ ions randomly occupy lattice sites.^38^ This disorder creates a symmetry break that triggers intensified emissions. In contrast, the cubic phase possesses larger symmetries. Studies reveal that samples containing both phases experience an increase in decay time with decreasing cubic phase content. The disparity in lifetimes can also be attributed to the prevalence of the hexagonal phase in samples obtained in the presence of the fluorinating agent.^31,39^ The obtained decay times are larger than those reported previously, indicating enormous potential for the development of photonics devices.^40^

**Figure 7:**
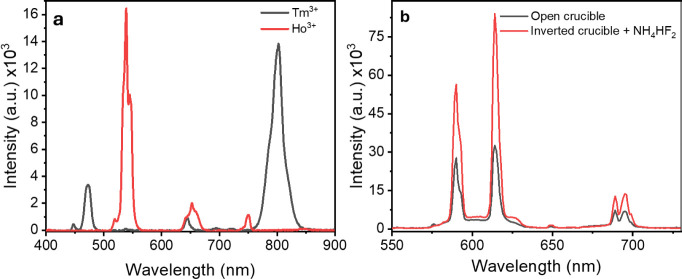
(a) Upconverting emission spectra of hexagonal NaYF_4_:Yb^3+^,Ho^3+^ and NaYF_4_: Yb^3+^,Tm^3+^ under NIR-975 nm excitation and (b) downconversion emission spectra of NaYF_4_:Eu^3+^ under UV-395 nm, both of them synthesized with the inverted-crucible device in the presence of NH_4_HF_2_ fluorinating agent.

To demonstrate further versatility of the method, hexagonal NaYF_4_:Yb^3+^,Ho^3+^ and NaYF_4_: Yb^3+^,Tm^3+^ upconverting particles were also synthesized through the inverted-crucible approach in the presence of NH_4_HF_2_, and the results demonstrated the expected luminescence properties under 975 nm, as shown in Figure 7a. In addition, a NaYF_4_:Eu^3+^ sample was synthesized, and its emission spectrum under 395 nm UV-light excitation is presented in Figure 7b. Therefore, this work demonstrates the synthesis of various lanthanide-doped materials capable of covering emissions in different regions of the electromagnetic spectrum through both up- and downconversion mechanisms.

## 4 Conclusions

The innovative method reported herein introduces a new paradigm for producing grams of pure-hexagonal NaLnF_4_ submicrometric particles. It requires instrumentation as simple as a standard hot plate, eliminating the need for an inert atmosphere, furnaces, or special reactors. To optimize the sintering process, three inverted crucibles were stacked in increasing size in an inverted-crucible scheme, which shifts the equilibrium reaction toward the generation of β-NaLnF_4_ particles. This system effectively enhances the heat transfer mechanism via convection, resulting in a faster temperature increase. Furthermore, the physical barrier hinders the free exchange of oxygen with the surrounding atmosphere, thus preventing crystal impurities that disrupt the effectiveness of upconversion frequency due to multiphoton relaxation. The synergy of implementing the inverted-crucible array with the incorporation of fluorinating agents such as NH_4_HF_2_ demonstrated a significant impact on reducing the heating temperature and the sintering time to 350 °C and 30 min, respectively. This promotes the integration of the precursors in the molten phase, thus enhancing the homogeneity, crystallinity, and upconverting emission intensity of the products. As additional advantages, the reported method allows for particle-size modulation at the submicrometric scale as a function of the sintering temperature and heating time, avoids the use of organic solvents for separation and washing, and provides quantitative reaction yields in the tens-of-grams scale. This was demonstrated by synthesizing a 20-g batch, demonstrating a broad advancement in the large-scale production of upconverting particles with further applications in a plethora of advanced materials.
